# Supplementary material for: A methodology for the design of experiments in computational intelligence with multiple regression models
Source: PeerJ. 2016 Dec 1;4:e2721. doi: 10.7717/peerj.2721 (PMC5136129; doi:10.7717/peerj.2721)
Supplement: Data S1 — Datailed results from UC Irvine Machine Learning Repository (Housing, Machine CPU, Wine Quality, Automobile and Parkinson) and the 3 Use Cases (Protein Corona, Gajewicz Metal Oxides and Aquatic Toxicity) [file peerj-04-2721-s001.zip › 6_UseCases/1_ProteinCorona/RFRFE.details.csv.repeatedcv.split9.pdf]

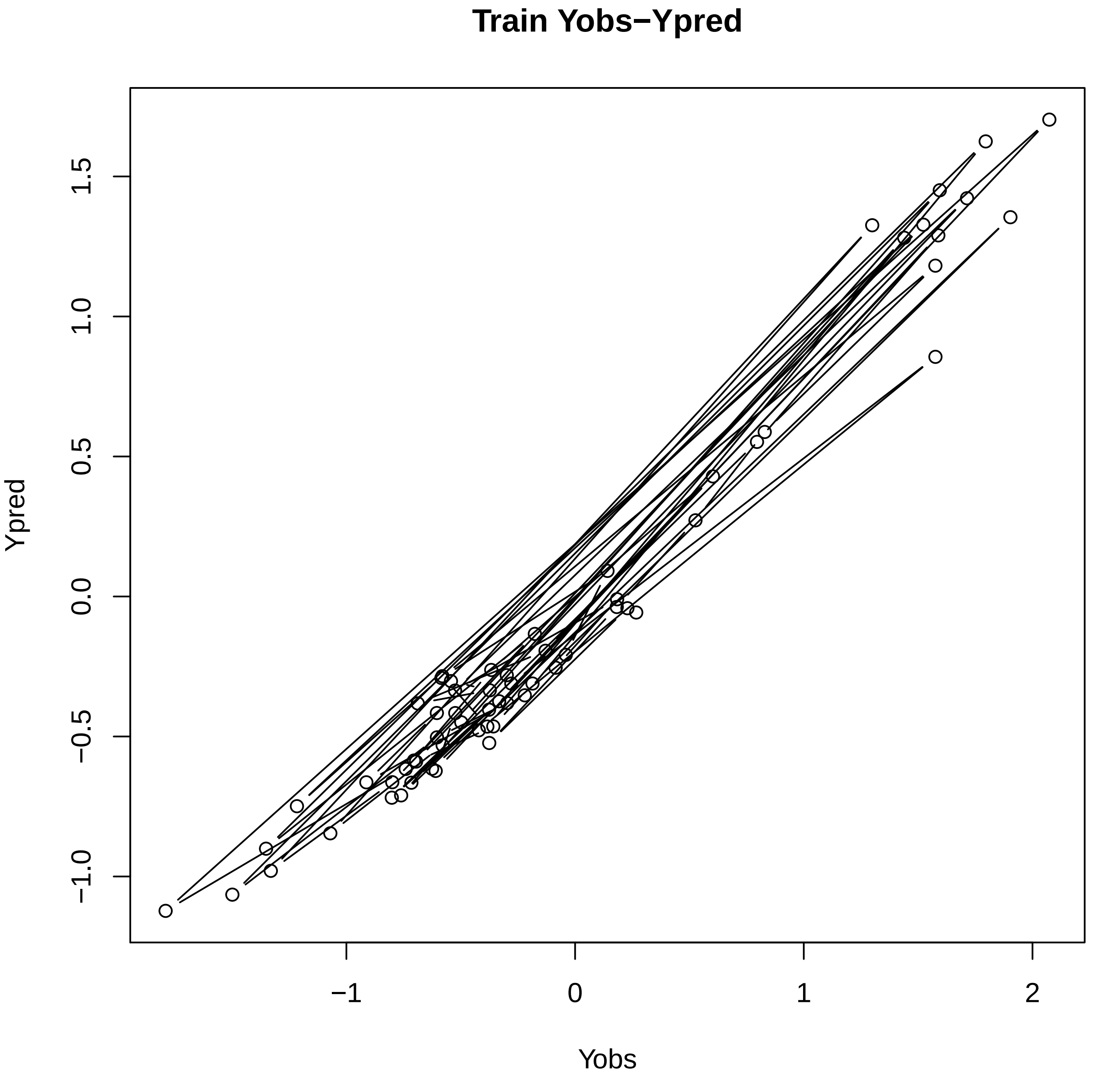
Train Yobs−Ypred
Ypred
Yobs
−1.0
−0.5
0.0
0.5
1.0
1.5
−1
0
1
2

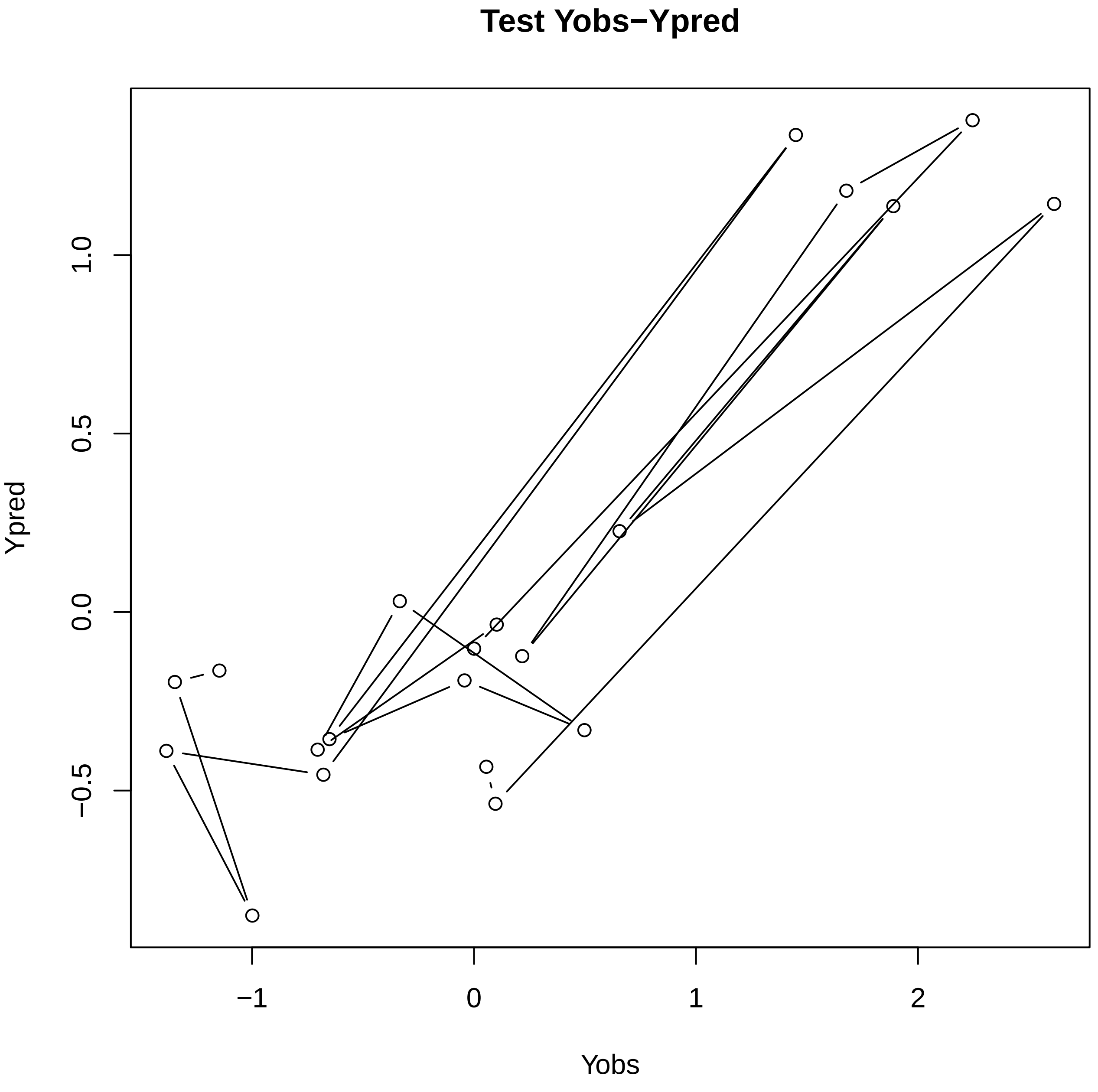
Test Yobs−Ypred
Ypred
Yobs
1.0
0.5
0.0
−0.5
−1
0
1
2

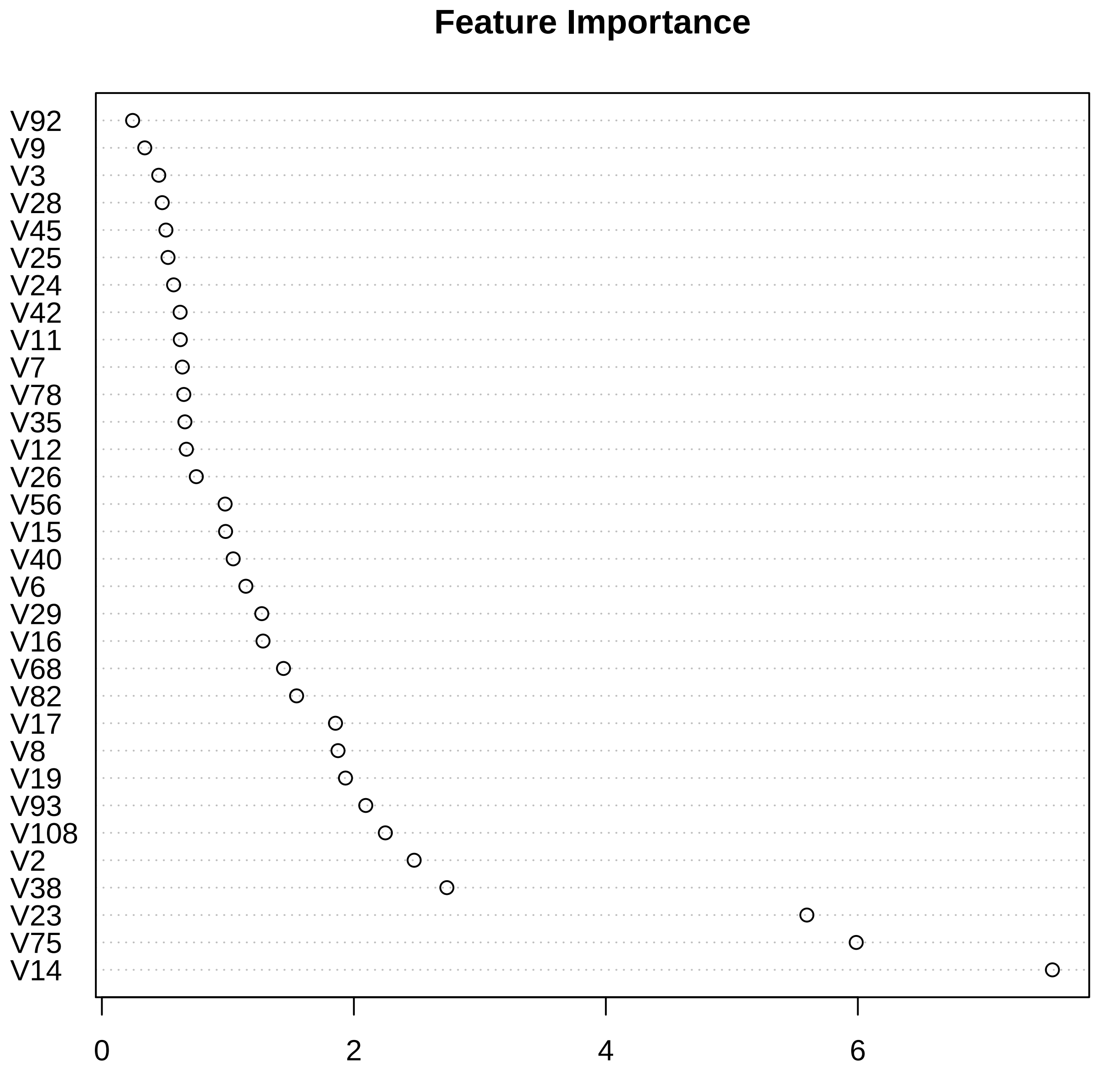
Feature Importance
V92
V9
V3
V28
V45
V25
V24
V42
V11
V7
V78
V35
V12
V26
V56
V15
V40
V6
V29
V16
V68
V82
V17
V8
V19
V93
V108
V2
V38
V23
V75
V14
0
2
4
6

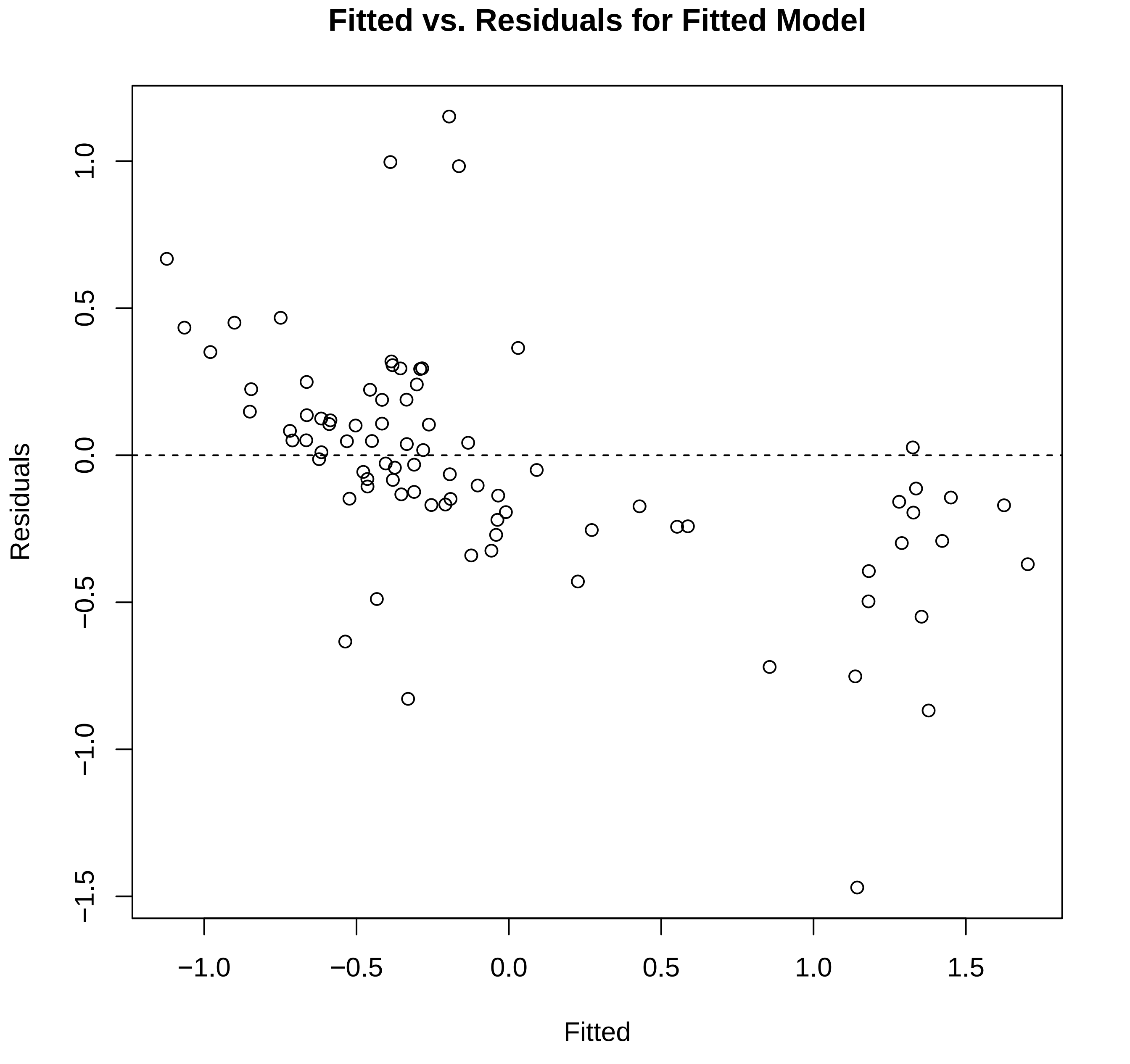

Fitted vs. Residuals for Fitted Model
Residuals
Fitted
1.0
0.5
0.0
−0.5
−1.0
−1.5
−1.0
−0.5
0.0
0.5
1.0
1.5
